# Ankylosing spondylitis

**DOI:** 10.4103/0975-9476.72619

**Published:** 2010

**Authors:** Mukesh Edavalath

**Affiliations:** *Government Ayurveda Hospital, Madikai, Kerala, India*

**Keywords:** Amavatha, ankylosing spondylitis, *HLA B27*, niruha basthi

## Abstract

Ankylosing spondylitis belongs to a group of rheumatic diseases known as the spondyloarthropathies (SpA), which show a strong association with the genetic marker *HLA-B27*. Inflammatory back pain and stiffness are prominent early in the disease, whereas chronic, aggressive disease may produce pain and marked axial immobility or deformity. Modern medicine has no established treatment for it. From the Ayurvedic perspective, the disease can fall under *amavata*, which may be effectively managed when intervention is started in its early stages. Niruha basthi with *Balaguduchyadi yoga*, combined by Shamana treatment with Rasnerandadi kwatha and Simhanada guggulu have been found effective in curbing its progression. This article presents a single case report in which these treatments achieved considerable success.

## INTRODUCTION

In modern medicine, ankylosing spondylitis (AS) is a chronic, systemic, inflammatory, rheumatic disorder of uncertain etiology primarily affecting the axial skeleton.[1,2] It usually starts in the late teens and early twenties and can lead to progressive bony fusion of the sacroiliac joints and the vertebral column; some patients may also show extra-articular manifestations.[1] In modern medicine, long-term use of nonsteroidal anti-inflammatory drugs (NSAIDs) and a life-long program of appropriate regular exercises has been the mainstay of symptom control for almost six decades. Traditional disease-modifying anti-rheumatic drugs (DMARDs) used for rheumatoid arthritis (RA) are ineffective in the typical AS patient with disease limited to the axial skeleton, including hip and shoulder joints.[3] Regimented Ayurvedic intervention in the early stages of the illness can be highly beneficial in that further progression of the illness can be prevented. Here I present the case of a 21-year-old male patient, whose early diagnosis of AS permitted successful management according to Ayurvedic principles. Though initially bed-ridden due to severe pain, he returned to normal life.

## CASE HISTORY

A 21-year-old male patient, who had apparently been normal one and a half months previously, insidiously developed low back pain, particularly on the left side, which progressively worsened over the following two days. On the third day he was unable to get out of bed, and was taken to an orthopedic specialist who diagnosed sciatica. He was managed accordingly for a week, following which he developed both fever and pain in bilateral knee and shoulder joints. He was then referred to a higher center (a medical college) for further evaluation. After thorough examination, he was diagnosed as having ankylosing spondylitis. He discharged himself against medical advice and presented to my outpatient department with the following complaints: severe pain in both the low back and bilateral knee joints, along with morning stiffness for more than 1 hour, and intermittent fever with associated chills and head ache. His low back pain radiated to the left lower limb. It was more during the morning and evening hours, subsiding in the middle of the day. There was no history of other constitutional features like vomiting, abdominal pain, or skin rashes, nor of trauma or other major medical or surgical conditions. The patient’s appetite was greatly reduced and was accompanied by constipation. Urine was passed without difficulty or burning sensation, but sleep was disturbed by the combination of pain and fever.

### Family history

The patient’s family had a history of polyarthritis. His 26-year-old uncle was suffering from AS, while his 62-year-old grandfather had been under treatment for rheumatoid arthritis for twenty years. The patient had been prescribed the following medications: rabeprazole, 20 mg once daily, indomethacin, 75 mg twice daily, and prednisolone, 60mg once daily.

### Examination

Vitals – pulse 86/min, regular, full volume, BP 130/82 mmHg (right arm sitting), temperature 99.4°F (oral, 9 am), and respiratory rate – 22/min. The nervous system, cardio-vascular system, and respiratory system were within normal limits (WNL). Per abdomen examination was normal except for liver which was palpable two fingers below right costal margin.

Spine – mild scoliosis was observed in the thoracolumbar region toward left, lumbar lordosis obliterated, and tenderness over L3, L4, L5 region, also tenderness over bilateral sacroiliac joints. Other joints – there was swelling, temperature, and tenderness over bilateral knee joints and tenderness in the left hip. Movements were restricted and painful. SLR (straight leg raising test) was positive on left. The investigations had the following findings. Blood Hb 10.6 g/dl, ESR 140mm/h, TC 15,300. DC: N 79%, L 18%, E 2%, B 01%. random blood sugar 110 mg/dl, CPK 138 U/L, serum creatinine 1.0mg/dl. Widal test – negative. Human leukocyte antigen (HLA) – B27 by flow cytometry – positive. HLA B27 by PCR (polymerase chain reaction) – detected. Urine examination was within normal limits except for pus cells 4–5/HPF. USG abdomen – mild hepatosplenomegaly was noted. X-ray LS spine revealed bilateral sacroilitis (grade 2) and obliteration of lumbar lordosis. MRI lumbar spine revealed altered marrow signal (hypo-intense on T1 and hyper-intense on T2 weighted MRI) involving left sacral ala and iliac bones adjacent to sacro-iliac joints indicating bilateral sacroilitis, more on the left.

The patient was thoroughly analyzed according to Ayurvedic norms, from which, by applying the method of exclusion, he was diagnosed as having Amavata[4] (see Discussion) and a treatment strategy was formulated. The *vyadhi* (disease) was considered *yapya*[5] (treatable). The patient’s parents were accordingly counseled regarding the nature of the illness and treatment was then begun.

### Treatment

Initially the patient was administered treatment for his fever (*Jwara chikitsa*),[5] as fever was one of the main presentations. *Amrithothara kwatha*[6] 60ml thrice daily, *Amavatari ras*[7] tablet (tab) 250 mg thrice daily, and *Amrutharishta*[6] 30ml twice daily were advised. Gradually over a period of 1 week, the modern medicines were tapered and stopped. Even after 2 weeks of the above medications, his fever did not subside, and he continued to have high temperatures particularly at night. Accordingly, *Amrutharishta* was replaced by *Chitrakasava*,[7] and *Amavathari ras* by *Vettumaran gulika*.[6] Slowly the fever started subsiding. When the fever had gone, *Lepa*[5] (external application of medicated paste) was applied to the sacroiliac and other painful joints together with a heated mixture of *kottamchukkadi choorna*[6] and *dhanyamla*.[6] Swelling in the knee joint subsided, but the patient started developing skin rashes, so the *lepa* was changed to *Grihadhoomadi*.[6] In this way, he was managed for a period of 1 month, after which he was able to walk without support, and the severity of the pain reduced. Also, after the fever had subsided, internal medication was changed to *Indukantham kwatha*[6] 15ml thrice daily, *Marma gulika*[6] 1 twice daily, *Shaddharana choorna*[4] 5g twice daily, while externally, *Rooksha Pinda Sweda*[5] (sudation with medicated bolus) with *Kolakulathadi choorna*[5] was applied for seven days. Since the *sweda* only brought the patient slight relief, treatment was extended to 10 days, after which he started feeling lightness (*laghava*) in the body. He was then given purgation (*Virechana*) with *gandharva eranda*[6] (Roots of Ricinus communis processed in castor oil.) on alternate days for one week, after which his appetite returned.

During this whole time period, the patient’s diet was restricted strictly to rice gruel, *yavagu*,[5] processed with *panchakola churna*,[7] with cooked green leafy vegetables with minimal salt and oil. *Mudga yusha* (green gram soup) was allowed once *amavathari rasa* was stopped. As his appetite improved, the patient was allowed to take easily digestible steamed food items. After the *Swedana* (sudation), the patient had good relief from the low back pain, and that in other joints, and was only bothered by the morning stiffness, which by then had reduced to half an hour. Accordingly *Niruha Basthi*[5] (medicated enema) was planned with modified *Balaguduchyadi yoga*[5] mentioned for *vata vikara* (treatment of *vata*) [Table 1].

**Table 1:** Contents of Balaguduchyadi basthi

Dravya	Contents	Quantity
Makshika	Honey	200ml
Lavana	Saindhava (rock salt)	15g
Sneha	Sahacharadi thaila[6]	200ml
Kalka	Paste[5] prepared out of Yavani (*Trachyspermum ammi L*), Madanaphala (*Randia spinosa*), Bilwa (*Aegle marmelos*), Kushta (*Saussurea lappa*), Vacha (*Acorus calamus*), Sathahwa (*Anethum sowa*), Musta (*Cyperus rotundus*), and Pippali (*Piper longum*).	40g
Kwatha	Decoction[5] prepared out of Bala (*Sida cordifolia L*), Amritha (*Tinospora cordifolia*), Thriphala (*Terminalia chebula, Phyllanthus embilica, Terminalia bellirica R*), Rasna (*Pluchea lanceolata*), Dashamoola (*Desmodium gangeticum, Uraria picta, Solanum indicum, Solanum xanthocarpum, Tribulus terrestris, Aegle marmelos, Clerodendron premnoses, Oroxylum indicum, Gmelina arborea, Pterospermum suaveolens*), Madanaphala (*Randia spinosa*)[8]	400ml

*Basti* (enema) was administered according to *kala basti*[5] format during which six *niruhas* (decoctions) and nine *anuvasanas* (oil) were administered. The medicine for *niruha basti* (decoction enema) was prepared by mixing the drugs in a mortar with pestle, adding component medicines in the following order: *makshika* (honey), *lavana* (salt), *sneha* (oil), *kalka* (paste), and *kwatha* (decoction). It was administered on an empty stomach, using a conventional *basti netra* (syringe) and rexine encase as *basti putaka* (bag). *Sahacharadi taila* was used for *anuvasana basti* (oil enema), the dose being 60ml, administered on alternate days after food. Following the course of *basti* (enema therapy), the patient experienced significant improvement in the pain and stiffness in his low back and other joints. Subsequently, the following *shamana*[5] (pacifying) medicines were given for a period of 2 months – *Rasnerandadi kwatha*[6] 15ml twice daily, *Simhanada guggulu*[7] one tablet thrice daily, and *Gandharva eranda* 15ml 6 am (empty stomach) once a week. As his Hb percentage was down to 9 mg%, *Ayaskrithi*[5] 30ml twice daily was also administered for a period of 1 month, which brought the Hb to 10.1mg%. Mild spinal exercises were also advised to prevent occurrence of stiffness in due course of time. [Table 2]

**Table 2:** List of medicines administered during the treatment course

Name of formulation	Dose	Duration	Rationale
Amrithothara kwatha[6]	60ml thrice daily	First 14 days	Jwara chikitsa
Amavathari ras tab[7]	250mg thrice daily	First 14 days	Jwara chikitsa
Amritharishta[6]	30ml thrice daily	First 14 days	Jwara chikitsa
Chitrakasava[7]	30ml thrice daily	15^th^ to 30^th^ day	Amavatha chikitsa
Vettumaran gulika[6]	One tab thrice daily	15^th^ to 30^th^ day	Jwara chikitsa
Indukantham kwatha[6]	15ml thrice daily	31^st^ to 48^th^ day	Jwara chikitsa
Marma gutika[6]	One tab twice daily	31^st^ to 48^th^ day	Amavatha chikitsa
Shaddharana choorna[4]	5gm twice daily	31^st^ to 48^th^ day	Amavatha chikitsa
Gandharva eranda[6]	15ml 6 am on alternate days	41^st^ to 48^th^ day	Virechana
Rasnerandadi kwatha[6]	15ml twice daily	49^th^ to 108^th^ day	Shamana
Simhanada guggulu[7]	One tab thrice daily	49^th^ to 108^th^ day	Shamana
Ayaskrithi[5]	30ml twice daily	49^th^ to 108^th^ day	Shamana

## RESULT

During initial stages of treatment, the patient had to endure increased amounts of pain due to the absence of pain killers. As he gradually started to improve, and once the fever had subsided, recovery was fast. After the course of *basti*, his appetite improved and he was totally relieved of pain. He was able to move his joints freely without stiffness and carry out his day-to-day activities. His ESR, which was initially 140/1st hour, had come down to 05/1st hour after 3 months of treatment. Considering the nature of the illness, even though the patient was free from complaints, chances of relapse were considerable.

## DISCUSSION

AS belongs to a group of rheumatic diseases known as spondyloarthropathies (SpA), which have a strong association with genetic marker HLA-B27.[1,2] AS usually develops in the second or third decade of life,[2] affecting young men more frequently than young women, the estimated male–female ratio ranging from 2.5 to 5:1.[9] The sacroiliac and hip joints are the most affected. The cervical spine is involved late in the disease. Other joints that may be involved include the ankles, wrists, shoulders, elbows, and small joints of the hands or feet. Morning stiffness and nocturnal back pain are hallmarks. Constitutional features (e.g., fever, anorexia, weight loss) are not uncommon at the onset. With progressive axial involvement, pain and stiffness result in difficulty in walking and other daily activities.

With regard to genetic marker, *HLA-B27*, linked with AS.[10] the actual risk of AS developing in an *HLA-B27*-positive person is estimated at 1–2%. But *HLA-B27* determination is seldom necessary to establish the diagnosis. Only in questionable cases without distinctive radiographic changes may the presence of *HLA-B27* be of diagnostic value. Lumbosacral spine X-ray can demonstrate sacroiliitis.[9] Axial radiographic findings also include marginal bridging syndesmophytes, interapophyseal joint fusion, and “squaring” of lumbar and thoracic vertebrae, collectively producing the classic appearance of a “bamboo spine.” Clinical course and disease severity are highly variable. Prolonged occurrence of the disease leads to ankylosis of the spine leading to kyphosis and other spinal abnormalities. Patients with AS are at risk of complications, some of which may be life-threatening like restrictive lung disease,[11] cauda-equina syndrome, post-traumatic intervertebral fractures, osteoporotic compression fractures, or spondylodiscitis.[12,13] The differential diagnosis for such a presentation includes collagen vascular diseases like rheumatoid arthritis, systemic lupus erythematoses, and also rheumatic fever.[1]

The first step in initializing Ayurvedic treatment is to arrive at as precise a diagnosis as possible based on its principles.[5] Rather than making an explicit correlation of Ayurveda’s classification of vyadhis (diseases) with those of modern medicine, it is always better to formulate an Ayurvedic diagnosis based on the presenting features of the particular patient. In the present case, the patient had *Katee shoola* (low back pain), *Ruja* in *janu* and *amsa sandhis* (pain in knee and shoulder joints), *Sopha* in *janu sandhis* (swelling in knee joints), *Jwara* (fever), *Aruchi* (loss of appetite), *Vibandha* (constipation), *Alasya* (lethargy), *Sthamba* (stiffness of body parts and joints), and *Gourava* (heaviness).[4,5] The pathologies considered for differential diagnosis within the Ayurvedic paradigm included *Jwara, Amavata, Vatarakta*, and *Gridrasi*.[4,5] Even though the patient had *jwara* (fever) as a feature, it was excluded because it was not the predominant presentation, which was pain in the low back and other major joints. Also *jwara* is an acute symptom, rather than chronic like AS, so in this context it could only be considered a symptom. Some of the presentations bore a similarity to *vatarakta*, but that was excluded by the absence of specific features of *rakta* (blood) involvement like *vaivarnya* (discoloration of skin), *kandu* (itching) and involvement of small joints of hands and feet. *Gridrasi* was similarly excluded by the patient having other unrelated features like *jwara, vibanda*, and pain in knee and shoulder joints. The patient had features of *ama*[5] (undigested toxic matter) in his body: *jwara* (fever), *vibanda* (constipation), *alasya* (lethargy), *gourava* (heaviness), *sopha* (swelling), and *aruchi* (loss of appetite). Along with this was the pain in *kati* (low back) and other *sandhis* (joints), all pointing toward the diagnosis of *amavata*.[4] N.B. we cannot generalize that every case of AS will have features of *amavata*, Ayurvedic diagnosis should always be based on presenting features. Consequently treatment was planned to first remove the *ama* (undigested matter) by improving digestion with *deepana*[5] (medicine), and digesting the *ama* with *pachana*[5] ones. Later, *Niruha basthi* (decoction enema) was administered as the principal treatment for *Vata dosha*. In due course, *shamana* (pacifying) medicines were advised to prevent relapse and improve the general health of the patient.

## CONCLUSION

This case highlights the fact that confidence can be placed in Ayurvedic treatment principles even in cases where modern medicine’s prognosis is poor. The patient was diagnosed in Ayurvedic terms and treated accordingly. On this basis, the *vyadhi* (disease) was identified as being *yapya*[5] (treatable), and treatment was planned accordingly. *Niruha basthi* (*madhutailika*) forms the mainstay of treatment in cases of rheumatic diseases provided it is administered at the right stages of the illness. Even though the claim cannot be made that the patient is completely cured of the illness, as of now he is symptomatically normal, which in the context of modern medicine is tantamount to returned to health, though this is not true in Ayurveda. Moreover, according to Ayurveda, future exacerbation and relapse can be prevented by proper diet and continuing medication. Further clinical studies should be conducted to validate the treatment principles applied in this case.
